# Physicochemical Properties of Runner Bean and Their Starch, With a Comparison to Corn Starch

**DOI:** 10.1111/1750-3841.70440

**Published:** 2025-07-24

**Authors:** Folasade E. Akinwumi, Bukola A. Onarinde, Sumit Konar, Nick Tucker, Samson A. Oyeyinka

**Affiliations:** ^1^ National Centre for Food Manufacturing, School of Agri‐Food Technology and Manufacturing University of Lincoln Holbeach UK; ^2^ School of Chemistry, Joseph Banks Laboratories University of Lincoln Lincoln UK; ^3^ School of Engineering and Physical Sciences University of Lincoln Lincoln UK; ^4^ School of Engineering Sciences, Separation Science Department LUT University Lahti Finland; ^5^ Centre for Innovative Food Research (CIFR), Department of Biotechnology and Food Technology, Faculty of Science University of Johannesburg Johannesburg South Africa

**Keywords:** functional properties, legume starch characterization, *Phaseolus coccineus*, physicochemical properties, runner bean starch

## Abstract

The growing demand for sustainable and plant‐based ingredients in the food and non‐food industries has driven interest in alternative starch and protein sources. Runner beans, a lesser known legume, offer a promising but underexplored source of starch with unique functional properties. This study aims to evaluate the physical, physicochemical, and functional properties of starch from two distinct varieties of runner beans (Scarlet Emperor and White Swan), which exhibit notable phenotypic differences in appearance. Corn starch was included as a reference sample for starch isolates. Scarlet Emperor starch exhibited significant differences in amylose, amylopectin, relative crystallinity, pasting, and flow properties but similar crystallinity (C‐type), oil absorption capacity compared to White Swan starch. Both starches also differed significantly from the corn starch control in terms of functional, pasting, and rheological properties. Extracted starches were pure with high starch content (∼96%), high lightness value (∼94) but different amylose content, 28.38 for White Swan and 30.89% for Scarlet Emperor compared to corn starch (29.67%). Both runner bean starches were smooth, bigger, and displayed higher viscosity and firmer gels than the corn starch control. These findings suggest that runner bean starch can complement with corn starch, providing better thickening capacity in food systems.

## 1 Introduction

Runner bean is an underutilized legume that is a rich in protein, similar to those found in other pulses such as Bambara groundnut (15%–27%) (Nwadi et al. 2020; A. T. Oyeyinka, Pillay, et al. 2017; S. A. Oyeyinka et al. 2018) and cowpea (16%–31%) (S. A. Oyeyinka et al. 2018; Rengadu et al. 2020a, 2020b). The beans also contain high levels of carbohydrates including starch, which may vary between 33.53% and 40.16% on dry matter basis (Piecyk et al. 2013). The protein and starch in runner beans represent valuable ingredients with potential applications in the industry. For example, the protein isolates from pulses have reportedly showed potentials as functional foods and nutraceuticals that could be used to manage high blood pressure and combat oxidative stress (Aluko 2015, 2021). On the other hand, the versatility of starch from pulses like runner beans makes it essential in manufacturing and processing applications such as a thickener and stabilizer in foods or as a binder and sizing in pharmaceutical and textile industry, respectively.

Despite the nutritional and potential applications of runner bean, the beans remain underutilized and poorly studied in several parts of the world (Mariscal‐Moreno et al. 2021). The underutilization could be due to regions of cultivation which limits awareness among consumers. The hard‐to‐cook defects typical of legumes, presence of antinutrients, and limited research to unlock the potentials of the beans may also explain the current low level of usage in food and industrial applications. With the growing demand for food ingredients with novel functionality, exploring runner bean as a potential source of protein and starch requires a knowledge of their physicochemical and functional properties (S. A. Oyeyinka, Singh, et al. 2017). In the United Kingdom, starch is mainly produced from wheat and potato which are staple crops, and other quantities needed are imported (de Bragança and Fowler 2004). Diverting staple crops for industrial starch production reduces their availability for direct human consumption, potentially driving up food prices. Thus, extracting starch from local crops such as runner beans represents a viable and sustainable option that not only minimizes the carbon footprint associated with importing starch but also promotes sustainable agriculture by utilizing locally adapted crops that require fewer inputs. This can provide additional income streams to local farmers, encouraging agricultural diversification, contributing to food security.

Previous studies on runner bean starch (Akinterinwa et al. 2014; Piecyk et al. 2013) are very limited, suggesting the need for more studies to unlock the potential of this crop. Using scanning electron microscopy and laser scattering particle size analyzer, Piecyk et al. (2013) revealed significant differences between the granule size (23.8–25.7 µm) of two runner bean varieties with both starches displaying oval or elliptical shapes. The amylose content of runner bean starches is moderately high (31.4%–32.6%) depending on the variety of the grain (Piecyk et al. 2013). Other studies on runner bean starch focused on the carboxymethylation of the native starch, with resulting enhanced swelling, solubility, water absorption but reduced pasting properties (Akinterinwa et al. 2014).

While some authors have reportedly showed that grain type influenced the physical and nutritional components of legumes (A. T Oyeyinka, Pillay et al. 2017), other researchers found no link between them (Kaptso et al. 2015). The knowledge of grain composition may be important for the purposes of nutrition and grain utilization (Amonsou et al. 2014). In this study, we hypothesize that runner beans would display different physical properties and chemical composition. Furthermore, grain type would influence starch physicochemical properties, and runner bean starch would display higher amylose content, higher peak viscosity, bigger granule size, and better functionality than the corn starch control. Hence, the aim of this research is to investigate the physical and chemical properties of the grain as well as pasting, functional, and physicochemical properties of the starch from two runner bean varieties.

## 2 Methodology

### 2.1 Materials

The runner bean (*Phaseolus coccineus*) varieties, Scarlet Emperor and White Swan used for the purpose of this research, were obtained from Elsom Seeds Ltd., Spalding, Lincolnshire, UK. According to the seed supplier, both runner bean varieties were cultivated under comparable agronomic and climatic conditions in the Netherlands. The corn starch used as reference sample and amylose were obtained from Sigma‐Aldrich (Poole, UK).

### 2.2 Color and Other Physical Properties of Grain

The tristimulus L* a* b* color parameters of the runner bean seeds were determined using a Chroma Meter CR‐400 (Konica Minolta, Japan), calibrated with a white standard before use. The seeds were placed in a transparent dish, and color readings were taken in triplicate from different randomly selected points. The same procedure was applied to the flour obtained from milling the seeds.

The physical properties of runner bean seeds, including length, width, and thickness, were measured using an electronic vernier caliper with an accuracy of 0.1 mm. Fifty seeds from each variety were measured in triplicate, and these dimensions were used to calculate the geometric mean diameter (*D*) (Equation 1), mean sphericity (𝝓) (Equation 2), and surface area (*S*) (Equation 3). The thousand‐seed weight was determined by weighing triplicate samples of a thousand seeds, and the average weight was recorded in grams (Bernardino‐Nicanor et al. 2017).

(1) $$D=\sqrt[{3}]{T.W.L},$$

(2) $$\phi =\frac{D}{L}\;,$$

(3) $$S=\pi D^{2}.$$

### 2.3 Proximate Analysis

The moisture (934.01), ash (923.03), and crude fat (920.39) contents of the flour obtained from the whole seeds were determined using standard methods of the Association of Official Analytical Chemists (AOAC 2006). Protein content was determined by multiplying the nitrogen content determined using Dumas's combustion method by 6.25. The total carbohydrate was calculated by the difference [100 − (moisture + ash + fat + protein)].

### 2.4 Starch Isolation

Starch extraction followed the wet method previously described (S. A. Oyeyinka et al. 2015). Briefly, the runner bean seeds were first dehulled by soaking them in water for 18 h using a seed‐to‐water ratio of 1:4. The seedcoats were manually removed before drying the seeds in a Binder hot air oven (Binder, Germany) at 50°C for 24 h. The dried, dehulled seeds were milled into flour using the waring blender (HGBTWTS3, Torrington, USA) and stored until needed for starch extraction. Flour was dispersed in distilled water at a ratio of 1:10, and the pH of the suspension was changed to 10 using NaOH (0.1 N) to solubilize proteins. The suspension was centrifuged using Eppendorf Centrifuge (5702R, Hamburg, Germany) to separate the solubilized proteins from sedimented starch. The obtained sediment was resuspended in water and passed through 250‐ and 90‐µm sieves sequentially to remove fibrous materials. The pH of the starch solution was adjusted to 7 using 1N HCl and then left undisturbed for a period of 3 h. The resulting starch was subjected to multiple water washes and subsequently dried in a Binder hot air oven at a temperature of 50°C. The dried starch was kept at a temperature of 4°C until it was needed for analysis. To assess the purity of the starch, the color and proximate composition of the starch were assessed as described in Sections 2.2 and 2.3, respectively.

### 2.5 Amylose Content

The amylose content of the runner bean starches was determined using the spectrophotometry method of Williams et al. (1970). In brief, 20 mg of the starch sample was dispersed in 10 mL of 0.5 N KOH. The suspension was homogenized for 5 min. It was then transferred to a 100‐mL volumetric flask and diluted to the calibration mark with distilled water. A 10‐mL aliquot of the starch solution was transferred into a 50‐mL volumetric flask, to which 5 mL of 0.1 N HCl and 0.5 mL of iodine reagent were then added. The volume was made up to 50 mL with distilled water, and the absorbance was read and recorded at 625 nm using a spectrophotometer (Jenway, 7305 Bibby Scientific, UK). A stock solution of pure amylose was prepared and subsequently diluted to generate working standards with absorbance values within the optimal range for spectrophotometric analysis. The standard curve was constructed using final amylose concentrations of ∼0.004, 0.008, 0.012, 0.016, and 0.020 mg mL^−1^. These concentrations yielded a linear relationship with an *R* ^2^ value of 0.9883, indicating good fit for the calibration curve.

### 2.6 Microscopy

The surface morphology of the starch samples was analyzed using a TESCAN VEGA scanning electron microscope equipped with a tungsten thermionic emission source. Imaging was performed at a landing energy of 3 keV and a beam current of 16 pA. Prior to imaging, starch samples were mounted onto aluminum stubs and sputter‐coated with a 5‐nm layer of gold to enhance conductivity and image quality. SEM micrographs were captured at magnifications of 2000× and 10,000× to effectively visualize the surface characteristics of the starch granules.

### 2.7 Particle Size

The particle size distribution of the starch samples was assessed using a Malvern Hydro Mastersizer 2000 (Malvern Panalytical, Worcester, UK). The starch samples were dispersed in water, creating a suspension that was pumped at a controlled speed into the laser chamber to ensure uniform dispersion. Particle size distribution was measured using laser diffraction, with results processed by the Mastersizer 2000 software. For each sample, Dv 0.1, Dv 0.5, and Dv 0.9 values were recorded.

### 2.8 Structural Characterization Using X‐Ray Diffraction and Fourier Transform Infrared Spectroscopy

Powder X‐ray diffraction (XRD) data were collected with a Bruker D8 Discover operating in reflection geometry with Cu‐Ka radiation (*λ* = 1.5406 Å). Data were acquired using coupled two‐theta/theta scans over a 2θ range of 5–40°, with a dwell time of 2 seconds per 0.016° step. The raw data were exported in the xy format and plotted on the OriginLab software. Wide‐angle XRD measurements were carried out using X‐ray diffractometer (Bruker D8 Discover, Cambridge, UK) equipped with Cu Kα radiation (*λ* = 1.5406 Å) in the 2‐theta range of 5°–30° at 40 kV, and an electric current of 40 mA. For FTIR spectra, starch samples were placed on the sample chamber, and the spectra were obtained in the transmittance mode from 500 to 4000 cm^−1^ after calibration. The mean of the scan was recorded to report the results for each spectrum.

### 2.9 Functional Properties of Starch

#### 2.9.1 Oil and Water Absorption Capacity

The oil and water absorption capacities of the starch from the two runner bean varieties were evaluated according to the methodology outlined by S. A. Oyeyinka et al. (2015), with slight modifications. To determine the oil absorption capacity, 1 g of each sample was transferred into a pre‐weighed 15‐mL centrifuge tube. Then, 10 mL of sunflower oil (density = 0.91 g L^−1^) was added, and the mixture was vortexed thoroughly to ensure even dispersion. The suspension was allowed to stand at room temperature (21°C) for 30 min before being centrifuged at 4500 rpm for 30 min using an Eppendorf Centrifuge (5702R, Hamburg, Germany). The supernatant was decanted, and the tube and its contents were reweighed. The oil absorption capacity was calculated by determining the increase in weight, expressed as grams of oil absorbed per gram of flour (g g^−1^). Water absorption capacity was measured using the same method: substituting oil for water.

#### 2.9.2 Swelling Power

The swelling power of the starch samples was assessed using the methodology described previously (Madruga et al. 2014; S. A. Oyeyinka et al. 2015). In brief, a suspension of 1 g of starch in 10 mL of distilled water was prepared in centrifuge tubes. The tubes were immersed in a water bath (Grant JBA12, UK) at varying temperatures of 50, 60, 70, 80, and 90°C, with continuous stirring for 30 min. After heating, the tubes were swiftly chilled in an ice bath and thereafter subjected to centrifugation using an Eppendorf Centrifuge (5702R, Hamburg, Germany) at a speed of 4500 rpm for 30 min. The supernatant was decanted, and the swelling power was calculated as the ratio of the weight of the swelled starch residue to the original dry weight of the starch sample.

#### 2.9.3 Syneresis

The percentage syneresis of the starch samples over time was determined using the method described by González et al. (2021). A starch suspension of 4% (w/w, dry basis) was prepared in a centrifuge tube and homogenized for 1 min using a vortex mixer. The starch suspension was then heated in a water bath (Grant JBA12, UK) at 95°C for 30 min, after which it was stored at 4°C for 48 h. At 0, 24, and 48 h, the heated starch suspension was centrifuged at 4500 rpm for 15 min using Eppendorf Centrifuge (5702R; Hamburg, Germany). The weight of the suspension before centrifugation and the weight of the exuded water (supernatant) after centrifugation were recorded. The percentage syneresis was calculated as the amount of water released from the starch gel as a percentage of the initial sample weight.

### 2.10 Pasting Properties Measurement

Pasting properties, including peak viscosity, trough viscosity, breakdown viscosity, setback viscosity, final viscosity, peak time, and pasting temperature, were determined using a Rapid Visco analyzer (RVA TecMaster; Perten Instruments, PerkinElmer, Bucks, UK) according to the standard method provided by the instrument. Starch samples (2.5 g, adjusted for moisture content) were mixed with a corresponding amount of distilled water to achieve the required consistency. The analysis was conducted using pre‐programmed heating and cooling cycles. All measurements were performed in triplicate, and results were expressed in centipoise (cP).

### 2.11 Rheology Measurement

The rheological properties of runner bean starch were measured using a Trios Rheometer (HR10 Malvern, UK), using a 25‐mm parallel plate with a gap size of 1000 µm at 25°C. The starch samples (4% w/v) were gelatinized at 95°C for 30 min in centrifuge tubes and cooled to room temperature. Steady shear rheological tests were performed at shear rates between 10^1^ and 10^3^ s^−1^. The data were fitted into a Power‐Law Model as follows:

(1) τ=K,

where *τ* is the shear stress (Pa), *k* is the consistency coefficient, (Pa.s) *n*, *γ* is the shear rate (s^−1^), and *n* is the flow behavior index.

### 2.12 Texture Characteristics

The starch prepared in the RVA was left in the canisters for day 0 and day 5 (stored at 4°C) to facilitate gelation. The gel formed (height: 2.5 cm and diameter: 3.5 cm) was evaluated for gel hardness using TA/XT2 texture analyzer (Stable MicroSystems, Surrey, UK). Each canister was placed upright on the metal plate, and the gel was compressed at a speed of 0.5 mm s^−1^ to a distance of 10 mm with a cylindrical plunger (diameter = 5 mm) as reported by Sandhu and Singh (2007).

### 2.13 Statistical Analyses

Starch samples were extracted in three replicates, and analyses were done in triplicate except for scanning electron microscope that was done in multiple times to get the best image possible. All data were averaged, and the results were reported as mean ± standard deviation. IBM SPSS statistics (version 27, New York, USA) was used to perform a one‐way analysis of variance on the data, while Duncan's test was used to determine significant differences among means at 5% probability. Additional statistical analysis was conducted using Pearson correlation in SPSS to examine the relationships between selected flour functional and pasting properties of the starches.

## 3 Results and Discussion

### 3.1 Color and Physical Characteristics of Runner Beans

The physical properties of runner beans such as thousand weight, seed length, width, thickness, geometric diameter, mean sphericity, and surface area are presented in Table S1. All parameters were similar between the two varieties, except for thousand seed weight, which was significantly higher in White Swan. Despite being heavier, White Swan seeds had a smaller surface area compared to Scarlet Emperor. These differences may be due to variation in seed moisture content, influenced by harvest timing or storage conditions such as temperature and relative humidity. Furthermore, the ash, protein, and fat contents were significantly different between the two cultivars, which likely contributed to the observed differences in thousand seed weight (Table S1). The average seed length recorded in this study (21.50 mm) is higher than the values previously reported for runner beans (7.80–15.7 mm) (Alcázar‐Valle et al. 2021; Bernardino‐Nicanor et al. 2017; Corzo‐Ríos et al. 2020; Palmero et al. 2011) and for other pulses such as Bambara groundnut (12.11–13.52 mm) (A. T. Oyeyinka, Pillay, et al. 2017), black‐eyed pea (9.19–9.47 mm) (Altuntas and Demirtola 2007), kidney bean (16.66–16.76 mm) (Altuntas and Demirtola 2007), and red gram seeds (Khan et al. 2020). Differences in seed dimensions may be linked to moisture variability, as demonstrated by Baryeh (2001), who found a positive correlation between grain moisture and linear dimensions in Bambara groundnut. The physical appearance of the runner beans showed that the two beans are different (Figure S1), further confirmed by color data, where White Swan showed substantially higher (approximately three times) lightness value than the Scarlet Emperor seeds. The difference in color and appearance could be associated with the inherent variation in chemical constituents such as phenolics (Barampama and Simard 1995; Boateng et al. 2008; A. T. Oyeyinka, Pillay, et al. 2017).

### 3.2 Proximate Composition of Runner Beans

The nutrient composition data on dry weight basis reveal that carbohydrates and proteins are the major macronutrients in runner beans (Table S1). While the carbohydrate content was similar between samples (average of 68%), other components, including protein, fat, ash, and moisture, were significantly different (*p* ≤ 0.05). Scarlet Emperor showed significantly higher protein content (26%) compared to White Swan (∼23%). However, White Swan beans exhibited significantly higher moisture, fat, and ash contents than Scarlet Emperor. The higher moisture content of White Swan may explain its greater thousand seed weight, as shown in Table S1. Furthermore, the fat content of the runner beans (average of 2.66%) was low, which confirms that the beans are pulses. According to the seed supplier, both Runner bean varieties were cultivated under the same agronomic and climatic conditions, suggesting that the observed variation among the seeds is primarily due to varietal differences. Pulses are dry beans with low fat content, usually less than 10% (S. A. Oyeyinka and Oyeyinka 2018). The observed nutrient composition of the two runner bean varieties is consistent with findings from other studies (Alcázar‐Valle et al. 2021; Bernardino‐Nicanor et al. 2017). Nevertheless, lower protein values (17.7%–18.0%) were reported elsewhere (Corzo‐Ríos et al. 2020), highlighting the impact of environmental conditions, soil quality, and genetic variation on the nutritional profiles of different bean varieties.

### 3.3 Starch Purity and Amylose Content

The extracted starches along with the control corn starch exhibited low levels of fat (0.14%–0.33%), ash (0.06%–0.10%) and protein (0.16%–0.39%), indicating a high degree of purity (Table 1). Runner bean starches had a lower moisture content (∼4%) compared to corn starch, which contained more than twice that amount (Table 1). This variation may be influenced by differences in drying and storage conditions, particularly since the runner bean starches were extracted and dried in the laboratory, while the corn starch was commercially sourced. Additionally, inherent differences in starch structure and composition between botanical sources may also contribute to moisture retention characteristics. The corn starch used in this study was obtained from Sigma‐Aldrich, while the runner bean starches were extracted and dried under laboratory conditions. The total starch content of the runner beans was ∼96%, significantly higher than that of the control corn starch (91.06%). This value also exceeds previously reported starch content for runner beans (92.20%–94.40%) by Piecyk et al. (2013), further confirming the purity of the extracted starches. The purity of the starches was further assessed by measuring the lightness value, which was very high (>90) for the three starches (Table 1). Starch color is a visual indicator of purity, and high values greater than 90 have been reportedly used to confirm starch purity in extracted starches (Vithu et al. 2020; H. Wang et al. 2020).

**TABLE 1:** Lightness, particle size, FTIR peak ratio, chemical composition, amylose content, functional, pasting, and gel firmness of runner bean and corn starches.

Properties	Scarlet Emperor	White Swan	Corn starch
Color and particle size			
L*	93.42 ± 0.23c	93.83 ± 0.71b	94.96 ± 0.39a
Dv 0.1 (µm)	15.07 ± 0.02a	15.79 ± 0.18b	6.31 ± 0.05c
Dv 0.5 (µm)	39.19 ± 17.32ab	28.61 ± 0.04a	18.16 ± 0.05b
Dv 0.9 (µm)	81.72 ± 2.67b	50.80 ± 0.11a	30.53 ± 0.06c
Starch composition			
Moisture (%)	4.01 ± 0.08b	4.01 ± 0.32b	8.84 ± 0.23a
Ratio of 1045/1022 cm^−1^	1.09 ± 0.01b	1.14 ± 0.02a	1.11 ± 0.02a
Ash (%)	0.10 ± 0.03a	0.06 ± 0.03a	0.10 ± 0.01a
Fat (%)	0.33 ± 0.57a	0.14 ± 0.24b	0.24 ± 0.05b
Protein (%)	0.39 ± 0.21a	0.16 ± 0.11b	ND
Total starch (%)	95.18 ± 0.54a	96.63 ± 0.41a	91.06 ± 0.22b
Amylose content (%)	30.89 ± 0.34a	28.38 ± 0.24c	29.67 ± 0.06b
Amylopectin (%)	69.11 ± 0.34c	71.62 ± 0.24a	70.33 ± 0.06b
Ratio of amylose/amylopectin	0.45 ± 0.01a	0.39 ± 0.01c	0.42 ± 0.00b
Relative crystallinity (%)	35.65 ± 0.12b	37.65 ± 0.04a	34.75 ± 0.20c
Functional properties			
Water absorption capacity (g/g)	1.08 ± 0.03a	1.08 ± 0.04a	0.66 ± 0.02b
Oil absorption capacity (g/g)	0.45 ± 0.02b	0.42 ± 0.01c	0.57 ± 0.01a
Flow behavior and pasting			
Consistency index‐K (Pa s*^n^*)	2.73 ± 1.15b	6.12 ± 1.22a	1.61 ± 0.13b
Flow behavior index‐*n*	0.33 ± 0.03b	0.28 ± 0.03c	0.49 ± 0.01a
*R* ^2^	0.98 ± 0.00ab	0.95 ± 0.02b	0.99 ± 0.00a
Pasting temperature (°C)	75.08 ± 0.08b	75.07 ± 0.03b	75.79 ± 1.10a
Peak viscosity (cP)	2983.67 ± 14.01a	2455.00 ± 13.53c	2729.56 ± 4.58b
Breakdown viscosity (cP)	1058.33 ± 13.50a	735.33 ± 16.26b	671.67 ± 1.53c
Final viscosity (cP)	3949.67 ± 10.02a	3572.33 ± 63.07b	3321.33 ± 5.86c
Setback viscosity (cP)	1994.33 ± 3.22a	1754.33 ± 15.01b	1259.33 ± 4.62c
Peak time (min)	4.33 ± 0.00b	4.38 ± 0.04b	5.42 ± 0.04a
Gel firmness			
Firmness at day 0	1255.58 ± 7.40b	1398.05 ± 75.73a	448.43 ± 37.06c
Firmness at day 5	4398.74 ± 245.10a	4036.70 ± 295.60a	1134.05 ± 135.08b

*Note*: Data are presented as mean ± standard deviation. Mean values with different superscript letters are significantly different (*p* ≤ 0.05).

Abbreviation: ND, not detected.

Corn starch showed intermediate amylose content (29.67%), compared to starch from White Swan (28.38%) and Scarlet Emperor (30.89%) when expressed on dry weight basis (Table 1). The amylopectin content of White Swan, calculated by difference, was the highest among the three starches. This observation is consistent with the fact that most starches are amylopectin‐rich, with varying proportions depending on the source. The amylose content in this study is lower than the values (average of 32%) reported for two runner varieties reported by Piecyk et al. (2013). This discrepancy may be due to genetic variation or environmental factors influencing amylose synthesis. The ratio of amylose to amylopectin in the starches followed this order: White Swan (0.39) < corn starch (0.42) < Scarlet Emperor (0.45), as seen in Table 1. This ratio is important as it directly impacts the functional behavior of the starch. For example, the amylose/amylopectin ratio provides a good indication of the potential textural and functional properties of starch‐based products. The higher amylose content in Scarlet Emperor starch (0.45 ratio) suggests it might form stronger gels (Table 1) and have higher viscosity (Table 1) under heating, making it more suitable for products requiring firmness and resistance to retrogradation, such as in baked goods.

### 3.4 Morphology and Particle Size Distribution

**FIGURE 1:**
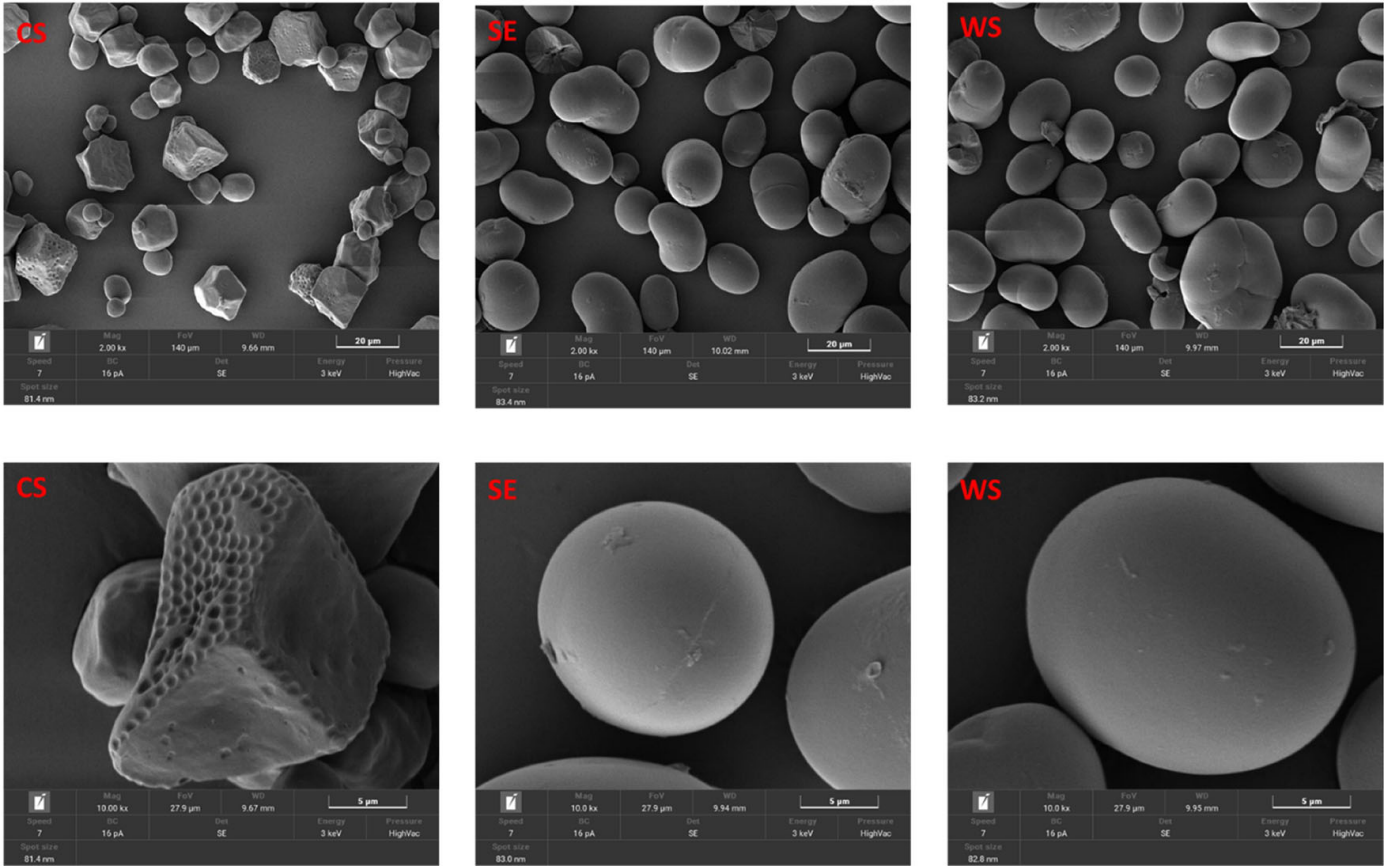
Scanning electron micrographs of corn and runner bean starches at 2000× and 10,000× magnifications. CS: corn starch; SE: Scarlet Emperor; WS: White Swan.

The micrographs of runner bean starches, which appear to be similar, show clear differences in the granule surface structure compared to the corn starch reference sample (Figure 1). Runner bean starch granules, which are predominantly rounded or oval with a smoother surface, were bigger in size (28.61–39.19 µm), in contrast to corn starch granules, which showed an average size of 18.16 µm (Table 1). The corn starch showed irregular‐to‐polygonal shapes with sharp edges and pore‐like structures, indicating that they are compound starches (Figure 1). Polygonal and irregularly shaped granules have been previously reported for corn (Flores‐Silva et al. 2017; B. Wang et al. 2022) and cocoyam or amadumbe starches (Mukurumbira et al. 2017; Naidoo et al. 2015; S. A. Oyeyinka and Amonsou 2020). The morphology of starch granules is a key factor in understanding the relationship between starch structure and its functional properties, playing a crucial role in the processing of starch and starch‐based materials (B. Wang et al. 2021). The porous structure in corn starch has several implications. For instance, it may increase digestibility by making the granules more accessible to digestive enzymes (Benavent‐Gil and Rosell 2017), while the smoother granules of runner bean starch are likely more resistant to enzymatic breakdown. Additionally, the porous nature of corn starch could improve its blending with polymers or additives, enhancing flexibility in bioplastics. In contrast, the smoother granules of runner bean starch may produce stronger, more rigid bioplastics, making them suitable for applications requiring greater mechanical strength (Li et al. 2015). The particle size analysis corroborates the morphological observations, indicating that runner bean starch granules (Scarlet Emperor and White Swan) are larger and exhibit greater heterogeneity than corn starch (Table 1). The Dv 0.1 values (∼15 µm) reflect larger minimum granule sizes in runner beans compared to corn starch (∼6 µm). Median particle sizes (Dv 0.5) ranged from 28.6 to 39.2 µm for runner beans, significantly exceeding the 18.2 µm median for corn starch. Furthermore, the Dv 0.9 values (50.8–81.7 µm) reveal a wider size distribution and increased variability in runner bean starches, consistent with their characteristic rounded, smooth granules. In contrast, corn starch granules are smaller and more irregular in shape. These findings align with previous reports indicating that legume starches typically have larger and more diverse granule size distributions compared to cereal starches (Bajaj et al. 2018).

### 3.5 FTIR and XRD

**FIGURE 2:**
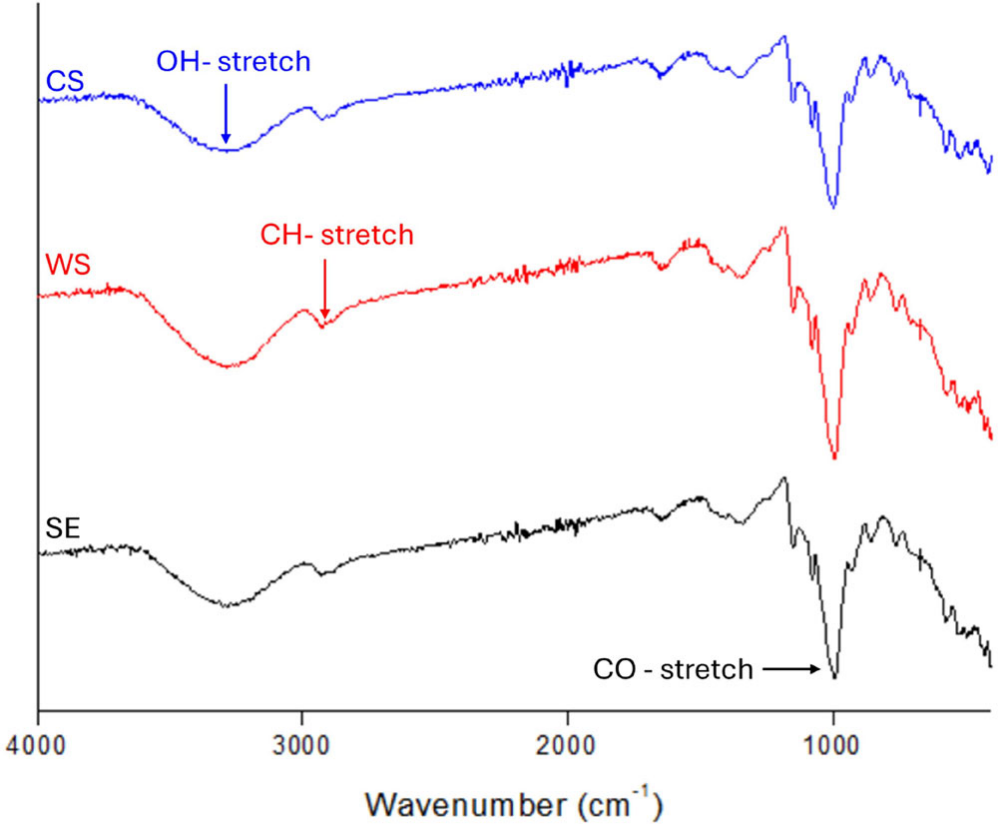
FTIR spectra of corn and runner bean starches. CS: corn starch; SE: Scarlet Emperor; WS: White Swan.

FTIR and XRD measurements provide information about functional group measurements and crystallinity in materials, and this information is important in explaining the functionality and changes in physicochemical properties of starch. The FTIR spectra of the three starch samples were identical, displaying typical starch structures with broad bands at around 3300 cm^−1^ corresponding to O─H stretch (Figure 2). The starch samples also showed typical peak around 2930 cm^−1^ attributable to C─H bond stretching (S. A. Oyeyinka et al. 2015), while a sharp peak in the fingerprint region, observed around 1000 cm^−1^ for all starch samples corresponds to C─O bond stretching (Estrada‐Leon et al. 2016). Scarlet Emperor starch showed a higher peak intensity than White Swan and corn starch which showed similar intensities in this C─H region. This could be attributed to variation in their amylose contents (Table 1). The peak corresponding to C─O stretch is reportedly influenced by the composition of starch (ratio of amylose to amylopectin) (Kizil et al. 2002). Previous studies also found variation in the intensities of peak in this region for starch isolated from various botanical sources such as potato, corn, wheat starch, waxy starch (Kizil et al. 2002), and Bambara starch (S. A. Oyeyinka et al. 2015). To further assess the differences in short‐range structure and crystallinity among the three starch isolates, the ratio of the bands at 1045 and 1022 cm^−1^, corresponding to the crystalline and amorphous regions of starch, respectively, was calculated from the spectra data and their average values reported in Table 1. Scarlet Emperor starch showed significantly lower value of 1.09 compared to White Swan and corn starches which showed high values. The lower ratio of 1045 and 1022 cm^−1^ in Scarlet Emperor starch agrees with the high amylose content (Table 1).

**FIGURE 3:**
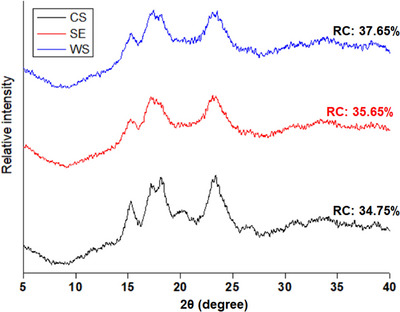
X‐ray diffraction patterns of corn and runner bean starches. CS: corn starch; RC: relative crystallinity; SE: Scarlet Emperor; WS: White Swan.

XRD patterns provided further insight into the crystalline polymorphs of the starches. The runner bean starches displayed a typical C‐type crystallinity pattern characterized by diffraction peaks at ∼15°, 17°, and 23° (2θ) (Figure 3). In contrast, the corn starch control exhibited an A‐type pattern with distinct peaks at ∼15°, a doublet at 17° and 18°, and a peak at 23° (2θ). The differentiation between A‐, B‐, and C‐type crystalline patterns relates to the packing arrangement of amylopectin double helices and hydration level within starch granules (Imberty and Perez 1988). The A‐type pattern corresponds to closely packed, less hydrated helices, whereas the B‐type pattern reflects a more hydrated core (Cheetham and Tao 1998; Imberty and Perez 1988). The C‐type polymorph, as observed here in runner bean starch, is generally regarded as a mixture of A‐ and B‐type arrangements, combining features of both packing density and hydration (Imberty and Perez 1988). Legume starches including those isolated from runner bean starch have been reported to display the C‐type polymorph (Hoover et al. 2010; Piecyk et al. 2013). These differences were supported by FTIR data, where the relative intensities of the 1045 and 1022 cm^−1^ peaks indicated variations in the crystalline and structural ordering (Table 1). The higher 1045/1022 ratio in White Swan and corn starches and their relatively lower amylose content agrees may explain differences in relative crystallinity values (34.75%–37.65%).

### 3.6 Functional, Rheological, and Gel Properties

#### 3.6.1 Water Absorption Capacity and Oil Absorption Capacity

The ability of runner bean starches to absorb water and oil was different from the corn starch control (Table 1). Both legume starches showed significantly higher water absorption capacity but lower oil absorption capacity than corn starch. The higher water absorption capacity of the runner bean starches may be attributed to their characteristic larger granule size (Figure 1 and Table 1). Larger granules have more surface area relative to their volume, providing increased sites for water interaction, especially in the amorphous regions where water absorption primarily occurs. Earlier researchers similarly found a positive correlation between bigger granules and high water absorption capacity (Bhat and Riar 2016). In addition, starches with high crystallinity such as the A‐type corn starch may exhibit lower water absorption because of the tight packing between the amorphous and crystalline domains as well as their intact granules which may restrict swelling.

Corn starch (57%) absorbed more oil than the two runner bean starches, which absorbed 42% and 45% oil for White Swan and Scarlet Emperor, respectively. This could be due to the smaller granule size and more angular morphology of corn starch which provide a greater surface area for oil binding. Runner bean starches showed smoother, more spherical granules, which are less efficient at trapping oil compared to the more irregular and porous corn starch (Figure 1).

#### 3.6.2 Swelling Power

**FIGURE 4:**
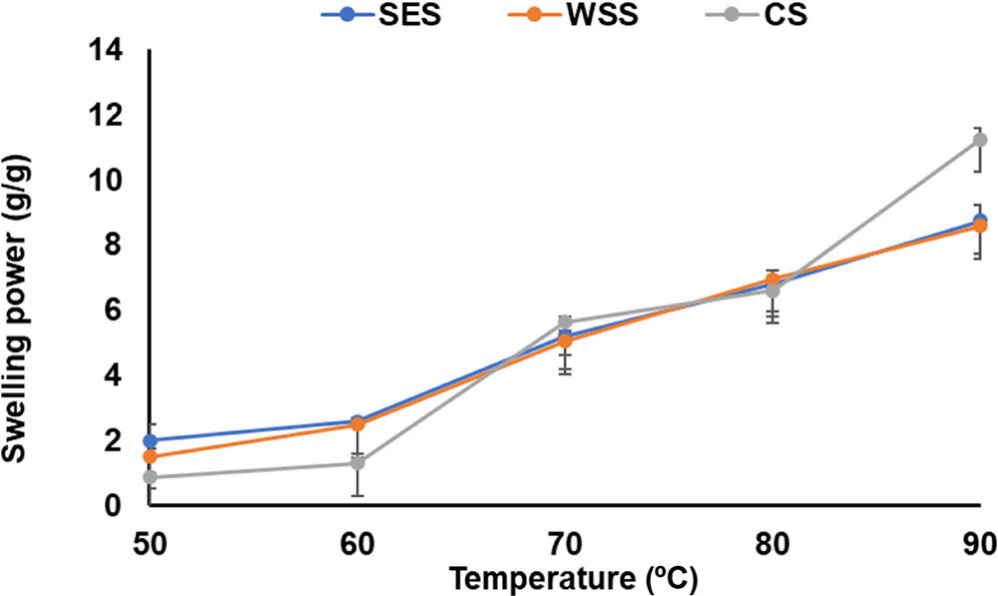
Swelling power of runner bean and corn starches. Error bars indicate standard deviation (N = 6).

The swelling power of the three starches increased with increasing temperature (Figure 4). Between 50 and 60°C, swelling power was progressively increasing, but a substantial increase was noticed between 70 and 90°C. The increase within this region has been associated with starch gelatinization. Furthermore, corn starch showed significantly higher swelling at 90°C compared with the legume starches, presumably due to its small granule size and porous surface. Tester and Morrison (1990) noted that amylopectin is mainly responsible for swelling, while amylose restricts swelling. This suggest that starches with higher amylose would display low swelling behavior. This was only true at 70 and 90°C, indicating that other factors can influence the swelling behavior of starches. A similar swelling pattern was reported for two runner bean starches by Piecyk et al. (2013). According to Singh et al. (2003), the magnitude of interaction between the amorphous and crystalline region between the starches may also influence the swelling behavior of starch from different botanical sources.

#### 3.6.3 Syneresis

**FIGURE 5:**
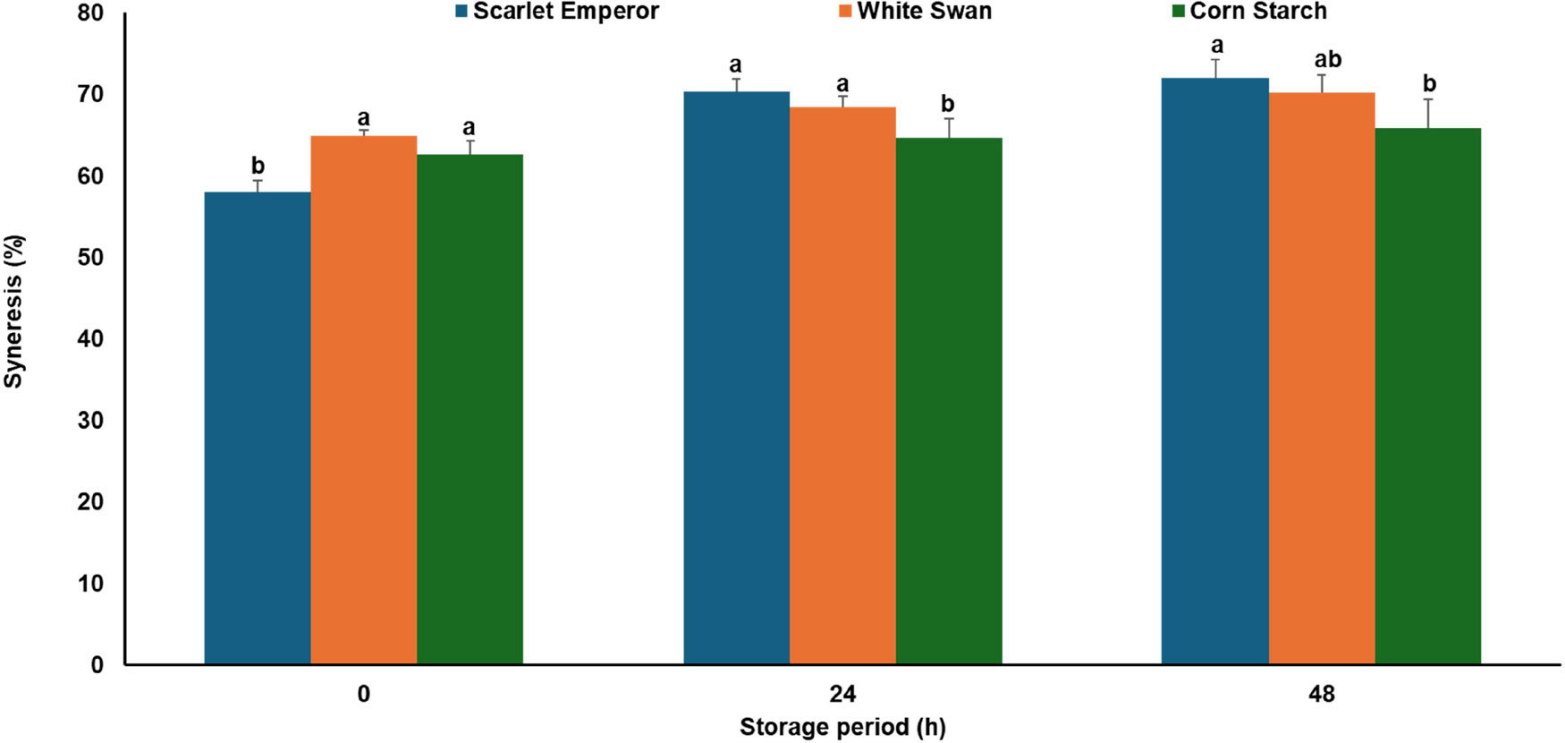
Syneresis of corn and runner bean starches. Error bars indicate standard deviation (N = 6). Different letters show a significant difference (*p* < 0.05).

Data for the release of water (syneresis) from freshly prepared and stored gels are shown in Figure 5. Syneresis refers to the separation of water from a gel matrix over time, caused by starch molecules (amylose and amylopectin) reorganizing into tighter structures. Initially, starch from Scarlet Emperor grain exhibited the least water separation (58%), followed by corn starch (63%) and White Swan starch (65%). Over time, water separation increased in all samples attributable to retrogradation. At 24 h, Scarlet Emperor (71%) surpassed White Swan (69%) and corn starch (65%), a trend that continued at 48 h (Figure 5). Higher amylose forms denser gels, which expel more water during molecular reassociation. This explains why runner bean starches exhibited higher syneresis than corn starch. Earlier studies found a significant positive correlation between big starch granules and syneresis (González et al. 2021). Thus, the higher syneresis of Scarlet Emperor may be attributed to its bigger granules (Figure 1 and Table 1). Large granules have a greater surface area and can absorb more water during gelatinization, leading to weaker gel structures that may release water more readily over time.

#### 3.6.4 Rheological Properties

The pasting profiles of the starch samples were similar (figure not shown), while the pasting properties are summarized in Table 1. These properties include pasting temperature, peak viscosity, breakdown viscosity, final viscosity, and setback viscosity, all of which play a crucial role in determining the functional characteristics of starch in various food applications. The pasting temperature, which represents the temperature at which the samples cook was very similar for the runner bean starches (∼75°C). These values were higher than the pasting temperature of ∼60°C previously reported for native and modified runner bean starches by Akinterinwa et al. (2014). On the other hand, starches from other *Phaseolus* species have been shown to have much higher pasting temperatures, ranging from 81.08 to 84.68°C (Ovando‐Martínez et al. 2011). When compared to corn starch (∼76°C), the runner bean starches displayed significantly lower pasting temperatures at *p* < 0.05 (Table 1), which could be due to differences in botanical source of the starch, granule size, and amylose content (S. A. Oyeyinka, Akinware, et al. 2021). Dreher and Berry (1983) noted that the small‐sized starch granules are more resistant to rupture and loss of molecular order during heating. Other factors such as the amylopectin structure in these starches may also influence the pasting temperature. Singh et al. (2009) found that pasting temperature showed a positive correlation with short amylopectin chains. Further studies on the chain length distribution of amylopectin in runner bean starches could help elucidate the role of this component in the pasting behavior and overall starch functionality.

The peak viscosity, which indicates the maximum swelling of starch granules during heating, was significantly higher in the Scarlet Emperor starch (2983.67 cP) compared to the White Swan starch (2455.00 cP) and corn starch (2729.56 cP). A higher peak viscosity suggests a greater ability of the starch to swell during pasting, which can result in thicker pastes. This difference in peak viscosity may be attributed to variations in starch composition, particularly lipid content, and amylose‐to‐amylopectin ratio. It is often expected that starches with higher amylose content will exhibit lower swelling due to amylose's ability to limit starch granule expansion (Naidoo et al. 2015). However, in this study, Scarlet Emperor, despite its higher amylose content (Table 1), exhibited a higher peak viscosity, indicating that factors other than amylose content, such as starch granule size or the presence of lipids, may also influence swelling. Granule size plays a crucial role in starch pasting behavior. The smaller granules of corn starch (Figure 1 and Table 1) may have contributed to its lower peak viscosity, as smaller granules typically absorb more water and swell faster because of an increased surface area (Nwaogazie et al. 2022). This suggests that the granule size distribution and surface characteristics of starches from different sources should be considered when evaluating their pasting properties.

Similarly, the breakdown viscosity, final viscosity, and setback viscosity of starch from Scarlet Emperor were significantly higher than those of White Swan and corn. High set back viscosity is generally associated with greater tendency to retrograde and has implications in food application, for example, in bread staling and noodles texture. The higher setback viscosity of Scarlet Emperor starch may be associated with its significantly higher amylose content compared to White Swan and corn starch (Table 1). Although amylose content plays a significant role in the retrogradation behavior of starch, other factors like the presence of a greater abundance of amylopectin with long chains results in a stronger retrogradation tendency (Kohyama et al. 2004), further indicating the need to investigate the chain length profile of amylopectin of runner bean starches in future studies. The high final viscosity of Scarlet Emperor starch indicates that it could provide a thick, stable consistency, making it ideal for products such as puddings, sauces, and gravies, where texture and mouthfeel are critical. In contrast, the lower setback, and final viscosities of White Swan and corn starches suggest that they may be more suitable for applications requiring moderate viscosity and less retrogradation, such as soups or gravies.

The apparent viscosity and rheological properties of corn and runner bean starches differ significantly, as reflected by their flow behavior index (*n*) and consistency index (*k*) (Table 1). The flow properties of the starches followed the Power‐Law model with the correlation coefficient (0.95 ≤ *R* ^2^ ≤ 0.99) close to 1. Runner bean starches generally exhibit higher viscosities compared to corn starches, likely due to differences in starch granule structure (Figure 1) and water‐binding capacity (Table 1). This is evident in the higher consistency index (*k*) of runner bean starches, indicating a thicker, more viscous solution at low shear rates (Table 1). While all three starches exhibit pseudoplastic (shear‐thinning) behavior, the differences in their flow behavior index (𝑛) and consistency index (𝑘) highlight the unique functional properties of these starches. The higher *n* value for corn starch compared to the runner bean starches suggest less structural disruption under shear which agrees with the pasting temperature result (Table 1).

#### 3.6.5 Gel Firmness

The firmness of gel prepared after pasting and storage for 0 and 5 days (Figure S2) revealed significant differences among the starch samples (Table 1). Runner bean starches exhibited higher initial gel firmness (1255.58 and 1398.05 g) compared to corn starch. This superior gel strength in the runner bean starches can be attributed to the structure of amylose in these starches. Legume starches predominantly show stronger gels than cereal starches due to the formation of dense three‐dimensional networks during gelatinization and cooling. The higher water absorption of the runner bean starch granules (Table 1) likely facilitated better hydration and network formation. After 5 days, the firmness of the gels increased substantially (153%–250%) for all the starches due to retrogradation. The variation could be attributed to amylose content and granule structure, with both playing crucial roles in gel formation. Starches with a more compact granule structure can lead to stronger gels as they can withstand higher degrees of swelling and maintain integrity during the gelling process. The irregular and porous nature of corn starch granules (Figure 1) may have resulted in the weaker gels compared to the runner bean starches.

### 3.7 Pearson Correlation Among Selected Structural, Functional, and Pasting Properties

The Pearson correlation analysis revealed suggestive relationships between starch composition, structure, and functional properties (Table S2). Amylose content showed a strong positive correlation with peak viscosity (*r* = 0.983, *p* < 0.05), indicating that starches with higher amylose levels develop greater viscosity during pasting. This suggests that amylose molecules contribute to the formation of a viscous network upon gelatinization, enhancing paste thickness. However, because only three starch sources were analyzed, the correlations should be interpreted with caution. The small sample size limits statistical confidence, and some relationships may simply be coincidental rather than reflecting true underlying mechanisms. While some studies report a significant negative correlation between amylose content and peak viscosity (Chung et al. 2011; Kayode et al. 2021; S. A. Oyeyinka, Oyedeji, et al. 2021), others have found no correlation (Hoover et al. 2010; S. A. Oyeyinka, Oyeyinka, et al. 2017) or even a positive relationship (Kong et al. 2015; Zhou et al. 2015). This variability suggests that factors beyond amylose content, such as amylopectin structure and granule morphology, also play important roles in determining peak viscosity. For example, Huang et al. (2007) attributed the higher peak viscosity of cowpea starch compared to chickpea and yellow pea starches to a greater proportion of long amylopectin chains. This finding aligns with Jane et al. (1999), who concluded that the chain length distribution of amylopectin, particularly its long branch chains, predominantly influences the pasting properties of starch. Furthermore, syneresis measured at 24 h (*r* = 0.892, *p* < 0.05) and 48 h (*r* = 0.813, *p* < 0.05) showed significant positive correlations with setback viscosity, indicating that starches with higher setback viscosity tend to retrograde faster. Syneresis also correlated positively with particle size at both time points, with Dv 0.1 (24 h: *r* = 0.794, *p* < 0.05; 48 h: *r* = 0.796, *p* < 0.05), Dv 0.5 (24 h: *r* = 0.598, *p* < 0.01; 48 h: *r* = 0.664, *p* < 0.01), and Dv 0.9 (24 h: *r* = 0.884, *p* < 0.05; 48 h: *r* = 0.710, *p* < 0.05). The Pearson correlation analysis also indicated that many functional and textural attributes were associated with particle size. For example, particle size showed significant positive correlations with hardness at days 0 and 5 (Table S2). This supports the observation that starch gels formed from larger granules tend to develop a firmer texture, likely due to a denser and more cohesive gel network. This agrees with previous studies where large granules showed greater hardness and higher water absorption (Bhat and Riar 2016). In this study, runner bean starches demonstrated a higher water absorption capacity than corn starch (Table 1), supporting the correlation data, which showed a significant positive relationship (0.687 ≤ *r* ≤ 0.957) between particle size and water absorption capacity (Table S2). However, the gels from larger granule also exhibited increased syneresis, suggesting that despite their firmness, they are more prone to water loss during storage. This apparent contradiction can be explained by the fact that hardness measures the gel's mechanical strength at a specific point, while syneresis reflects the gel's ability to retain water over time. It is important to note that correlations observed in this study should be interpreted in the context of limited sample diversity. Additional studies involving more starch sources would be necessary to confirm and generalize these findings.

## 4 Conclusion

This study assessed the composition of beans and determined the functionality of starch extracted from two runner bean varieties. Two varieties of runner bean studies exhibited very similar physical properties, except for thousand weight and total carbohydrate. Scarlet Emperor had a darker color, lower moisture content, and higher protein content, but White Swan was bigger. Both runner bean starch granules were smooth and bigger, and the gels were firmer than the corn starch control. Scarlet Emperor had higher amylose content and exhibited greater ability to absorb water, syneresis, swelling capacity, peak, breakdown, setback, and final viscosities than White Swan and corn starches, suggesting its suitability as a thickening agent in food products such as sauces, gravies, pie fillings, and pudding mixes. Overall, it appears that particle size plays a major role in influencing most of the functional and pasting properties of both runner bean and corn starches. Future studies should investigate the chain‐length distribution of amylopectin in the starches to better understand its impact on starch properties and explore a wider range of Runner bean cultivars to generate more robust data. Furthermore, the in vitro digestibility of native and cooked starches, modification of the starch with ligands, and their application in food systems is needed.

## Author Contributions

**Folasade E. Akinwumi**: conceptualization, investigation, methodology, validation, formal analysis, data curation. **Bukola A. Onarinde**: Conceptualization, investigation, funding acquisition, methodology, validation, visualization, formal analysis, supervision. **Sumit Konar**: Methodology, validation, visualization, formal analysis, software, project administration, data curation, resources. **Nick Tucker**: Investigation, methodology, writing – review and editing, validation, formal analysis, resources. **Samson A. Oyeyinka**: Conceptualization, investigation, funding acquisition, writing – original draft, writing – review and editing, visualization, methodology, validation, formal analysis, project administration, supervision, resources, data curation, software.

## Conflicts of Interest

The authors declare no conflicts of interest.

## Supporting information




**Supplementary Figures**: jfds70440‐sup‐0001‐Figures.docx

**Supplementary Tables**: jfds70440‐sup‐0002‐Tables.docx
