# Supplementary material for: Physicochemical Properties of Runner Bean and Their Starch, With a Comparison to Corn Starch
Source: J Food Sci. 2025 Jul 24;90(7):e70440. doi: 10.1111/1750-3841.70440 (PMC12287891; doi:10.1111/1750-3841.70440)
Supplement: Supplementary file 1 — Supplementary Figures: jfds70440‐sup‐0001‐Figures.docx [file JFDS-90-0-s002.docx]

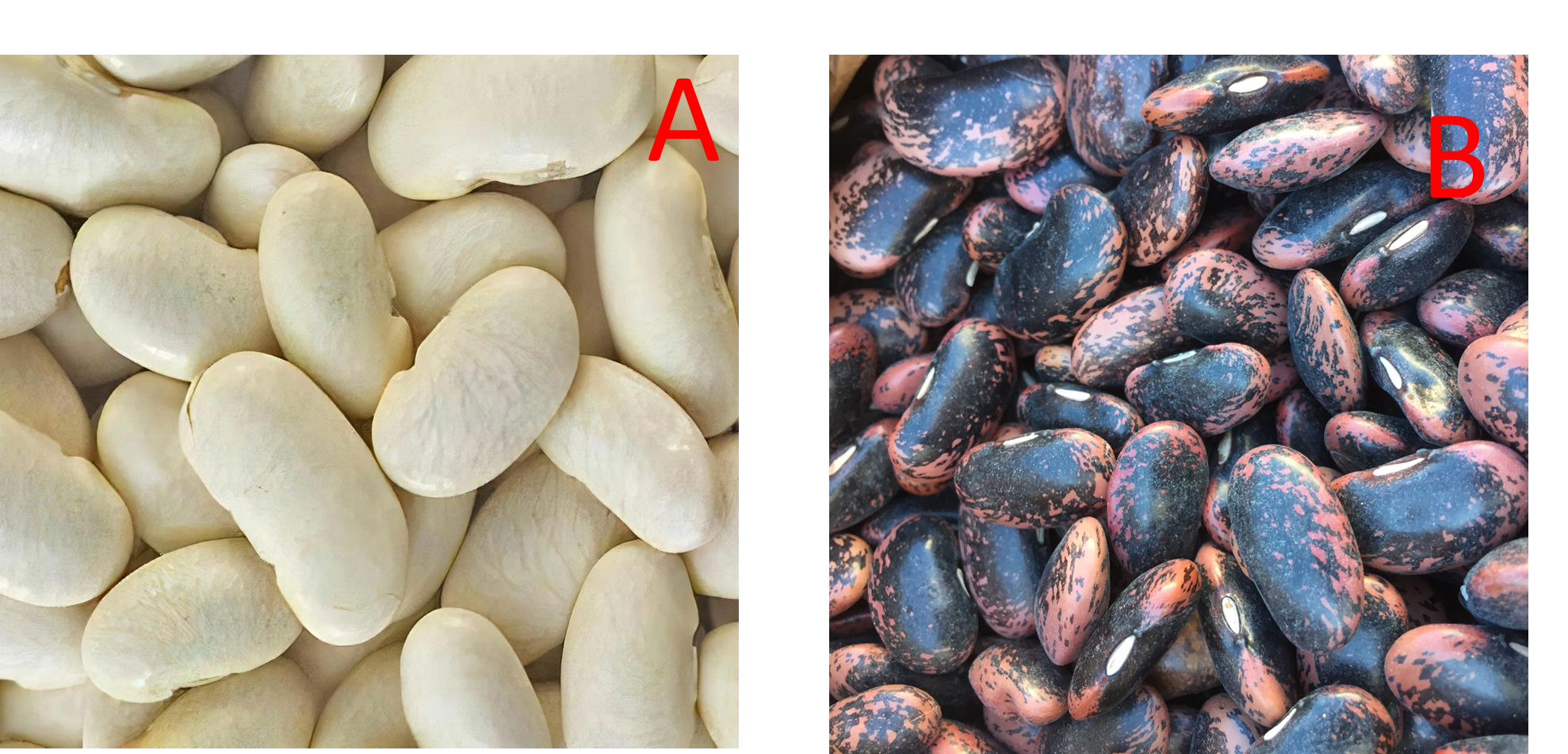


Fig. S1. Images of runner bean

A: White Swan; B: Scarlet Emperor


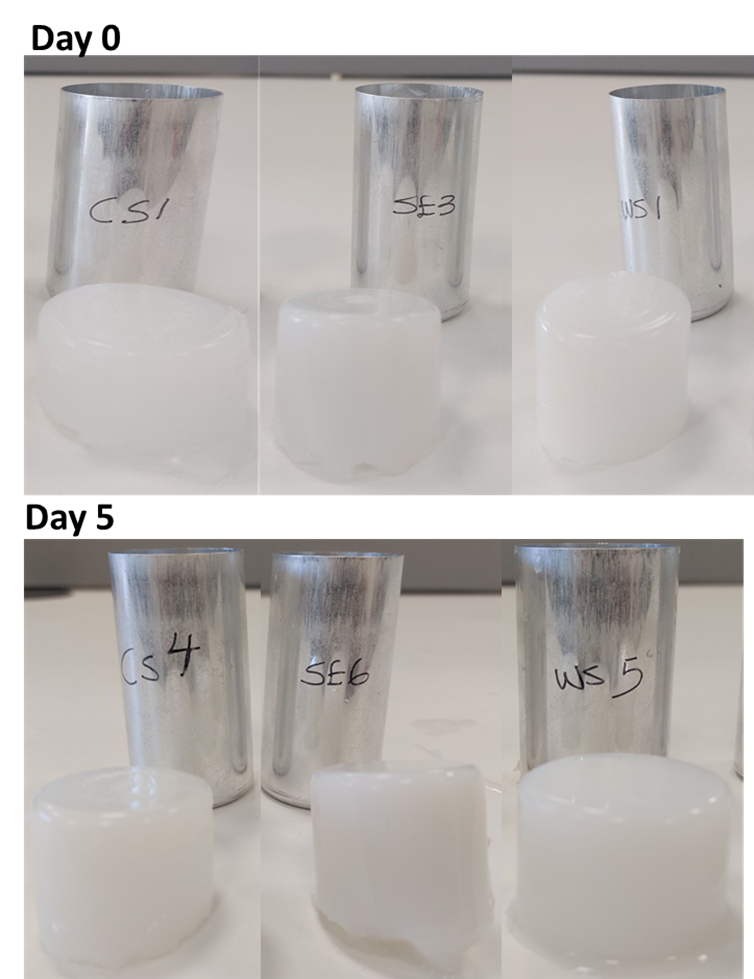


Fig. S2. Appearance of gels from pasted starch samples

CS1: Corn starch gel after day 0 CS4: Corn starch gel after day 5 SE3: Scarlet Emperor starch gel after day 0; SE6: Scarlet Emperor starch gel after day 5; WS1: White Swan starch gel after day 0; WS5: White Swan starch gel after day 0
