# Supplementary material for: Physicochemical Properties of Runner Bean and Their Starch, With a Comparison to Corn Starch
Source: J Food Sci. 2025 Jul 24;90(7):e70440. doi: 10.1111/1750-3841.70440 (PMC12287891; doi:10.1111/1750-3841.70440)
Supplement: Supplementary file 2 — Supplementary Tables: jfds70440‐sup‐0002‐Tables.docx [file JFDS-90-0-s001.docx]

Table S1

Physical and chemical properties of runner beans

| Parameters | Scarlet Emperor seed | White Swan seed |
| --- | --- | --- |
| Physical properties |  |  |
| Thousand Weight(g) | 1189.68±8.59^b^ | 1290.88±9.69^a^ |
| Seed Length L (mm) | 21.47±0.86^a^ | 21.54±0.10^a^ |
| Seed width W (mm) | 12.11±0.44^a^ | 11.49±0.34^a^ |
| Seed thickness T (mm) | 8.41±0.52^a^ | 8.36±0.40^a^ |
| Geometric diameter De (mm) | 12.98±0.59^a^ | 12.74±0.50^a^ |
| Mean sphericity (Φ) | 0.60±0.01^a^ | 0.59±0.01^a^ |
| Surface area S (mm^2^) | 529.51±47.37^a^ | 510.15±39.97^a^ |
| Colour |  |  |
| L* | 29.50±4.21^b^ | 84.58±4.15^a^ |
| a* | 9.84±1.00^a^ | 1.28±0.04^b^ |
| b* | 5.54±0.82^b^ | 16.40±0.93^a^ |
| Proximate composition dry weight basis (%) | |  |
| Moisture | 8.41±0.10^b^ | 11.25±0.09^a^ |
| Ash | 5.10±0.07^b^ | 5.39±0.09^a^ |
| Protein | 26.00±1.64^a^ | 22.89±1.08^b^ |
| Fat | 2.41±0.05^b^ | 2.90±0.16^a^ |
| Total carbohydrate | 66.48±1.70^a^ | 68.83±0.84^a^ |

^Data presented as mean ± standard deviation. Mean values with different superscript letters are significantly different (p ≤ 0.05) using independent samples T test.^

Table S2

Pearson Correlation Coefficients Among Structural, Functional, and Pasting Properties of Starch Samples

|  | Amylose | RC | Amp | SY0 | SY24 | SY48 | d 0.1 | d 0.5 | d 0.9 | HD5 | HD0 | PV | BV | FV | SV | PT | WAC |
| --- | --- | --- | --- | --- | --- | --- | --- | --- | --- | --- | --- | --- | --- | --- | --- | --- | --- |
| Amylose | 1 |  |  |  |  |  |  |  |  |  |  |  |  |  |  |  |  |
| RC | -.673^*^ | 1 |  |  |  |  |  |  |  |  |  |  |  |  |  |  |  |
| Amy | -1.000^**^ | .673^*^ | 1 |  |  |  |  |  |  |  |  |  |  |  |  |  |  |
| SY0 | -.912^**^ | .490 | .912^**^ | 1 |  |  |  |  |  |  |  |  |  |  |  |  |  |
| SY24 | .300 | .379 | -.300 | -.332 | 1 |  |  |  |  |  |  |  |  |  |  |  |  |
| SY48 | .131 | .549 | -.131 | -.195 | .698^*^ | 1 |  |  |  |  |  |  |  |  |  |  |  |
| d 0.1 | -.080 | .778^**^ | .080 | -.123 | .794^**^ | .796^**^ | 1 |  |  |  |  |  |  |  |  |  |  |
| d 0.5 | .302 | .292 | -.302 | -.288 | .598^*^ | .664^*^ | .616^*^ | 1 |  |  |  |  |  |  |  |  |  |
| d 0.9 | .577^*^ | .180 | -.577^*^ | -.680^*^ | .884^**^ | .710^**^ | .755^**^ | .658^*^ | 1 |  |  |  |  |  |  |  |  |
| HD5 | .079 | .670^*^ | -.079 | -.235 | .850^**^ | .823^**^ | .979^**^ | .711^**^ | .839^**^ | 1 |  |  |  |  |  |  |  |
| HD0 | -.143 | .809^**^ | .143 | -.063 | .768^**^ | .756^**^ | .994^**^ | .588^*^ | .712^**^ | .970^**^ | 1 |  |  |  |  |  |  |
| PV | .983^**^ | -.715^**^ | -.983^**^ | -.868^**^ | .310 | .087 | -.132 | .287 | .547 | .029 | -.191 | 1 |  |  |  |  |  |
| BV | .759^**^ | -.069 | -.759^**^ | -.795^**^ | .802^**^ | .585^*^ | .567 | .643^*^ | .965^**^ | .686^*^ | .516 | .739^**^ | 1 |  |  |  |  |
| FV | .569 | .192 | -.569 | -.660^*^ | .882^**^ | .718^**^ | .758^**^ | .707^*^ | .992^**^ | .854^**^ | .722^**^ | .538 | .957^**^ | 1 |  |  |  |
| SV | .298 | .484 | -.298 | -.454 | .892^**^ | .813^**^ | .925^**^ | .703^*^ | .946^**^ | .964^**^ | .895^**^ | .255 | .836^**^ | .944^**^ | 1 |  |  |
| PT | .023 | -.716^**^ | -.023 | .184 | -.840^**^ | -.735^**^ | -.970^**^ | -.613^*^ | -.775^**^ | -.948^**^ | -.954^**^ | .066 | -.600^*^ | -.773^**^ | -.920^**^ | 1 |  |
| WAC | .167 | .592^*^ | -.167 | -.352 | .863^**^ | .798^**^ | .957^**^ | .687^*^ | .883^**^ | .984^**^ | .943^**^ | .118 | .740^**^ | .898^**^ | .977^**^ | -.952^**^ | 1 |

Amp: Amylopectin; SY0: Syneresis at 0h; SY24: Syneresis at 24h; SY48: Syneresis at day 48h; d0.1: 10% of the particles are smaller than this diameter; d0.5 (Median): 50% of the particles are smaller than this diameter; d0.9: 90% of the particles are smaller than this diameter; HD5: Hardness at day 5; HD0: Hardness at day 0; PV: Peak viscosity; BV: Breakdown viscosity; FV: Final viscosity; SV: Setback viscosity; PT: Pasting temperature; s. * = Significant correlations at 10% (0.01); ** = Significant correlations at 5% (0.05)
